# Impact of the COVID-19 Pandemic on Blood Donation Patterns: A Systematic Review and Meta-Analysis

**DOI:** 10.7759/cureus.43384

**Published:** 2023-08-12

**Authors:** Abhay Kumar, Sushma Kumari, Usha Saroj, Ashwini Verma, Kumari Asha Kiran, Manoj Kumar Prasad, Rashmi Sinha, Mani Bhushan K Sinha

**Affiliations:** 1 Department of Internal Medicine, Rajendra Institute of Medical Sciences, Ranchi, IND; 2 Department of Blood Bank, Rajendra Institute of Medical Sciences, Ranchi, IND; 3 Department of Microbiology, Phulo Jhano Medical College, Dumka, IND; 4 Department of Preventive Medicine, Rajendra Institute of Medical Sciences, Ranchi, IND; 5 Department of Physiology, Rajendra Institute of Medical Sciences, Ranchi, IND

**Keywords:** covid-19, blood transfusion, blood donation, meta-analysis, systematic review

## Abstract

Blood centers, which are arguably the backbone of every hospital, depend on blood donors for a constant and regular supply of blood. Like many other fields, the COVID-19 pandemic severely affected blood donations. In this article, we aim to systematically search the studies done on blood donation during the COVID-19 pandemic period, analyze the pandemic’s effect on blood donation, and examine the methodology used to overcome the problem. We performed a systematic review and meta-analysis to investigate the effect of the COVID-19 pandemic on blood donation. Two independent reviewers searched different databases, such as PubMed, ProQuest, Scopus, and Google Scholar. We used the Preferred Reporting Items for Systematic Reviews and Meta-Analyses and the Joanna Briggs Institute critical appraisal checklist for overall study characteristics. We included a total of 15 studies. There was an overall decrease in blood donation of 25%, with some regions showing a decrease of as much as 71%. However, some regions were able to experience a 2-10% increase in blood donation after taking stringent and early measures to prevent such decreases. The COVID-19 pandemic and consequent lockdown greatly affected blood transfusion services, resulting in a progressive decline in blood donations that threatened the lives of many patients who were fully dependent on blood transfusion. However, by making appropriate and early decisions and taking action, policymakers and the rest of society can prevent such shortages, potentially saving millions of lives.

## Introduction and background

The COVID-19 pandemic severely impacted the lives of every human being. It affected every person in the world in one way or another. Likewise, it affected blood donation drives all over the world. Blood donation is a key component of every blood center. Blood availability is necessary for every hospital in the treatment of both medical and surgical patients, where blood transfusion is often a life-saving procedure. As COVID-19 was perceived by many to be a severe threat, people often chose not to come forward to donate blood, affecting blood donation drives globally. However, at that time, although blood demand was also decreased due to the postponement of planned non-emergency surgeries, shortages led to difficulties in supplying blood to people suffering from illnesses such as sickle cell anemia, thalassemia, as well as patients on hemodialysis, who require regular blood transfusions to survive.

In our study, we collected data systematically from studies done in all regions of the world, analyzed the factors that influenced blood donation, and searched for ways to increase blood donation in different regions of the world and maintain blood demand and supply. We hope that nations will be able to use our results to create stronger policies to increase blood donations and maintain an uncompromising blood supply to patients if similar pandemics occur in the future. The data were reviewed independently by two reviewers using predefined criteria. We searched relevant articles in PubMed, Medline, ProQuest, and Google Scholar published between January 2020 and June 2022. An earlier systematic review included only six studies [1]; therefore, we tried to do the study in a broader sense, including more studies from different regions of the world.

## Review

Methods

We registered the protocol for our systematic review and meta-analysis in the Prospero database (Registration No.: CRD42022308943). We followed the Preferred Reporting Items for Systematic Reviews and Meta-Analyses (PRISMA) guidelines for the selection of studies.

Study inclusion and exclusion criteria

We mainly took original articles containing data related to the impact of the COVID-19 pandemic on blood donation from January 2020 to June 2022 from retrospective and cross-sectional studies done across the world with data on blood donation done during the COVID-19 pandemic and the pre-pandemic period. We included articles showing comparative data in whole numbers only, before and during the pandemic. We excluded data showing blood donations per day instead of total numbers. We included only original articles. We also excluded letters to the editor, editorials, reviews, brief reports, and supplementary articles. We included only articles published in English.

PICO criteria

We defined the research question by employing the PICO device. Patients (P) were all healthy blood donors who fulfilled the criteria of blood donation defined by the World Health Organization (WHO). Exposure (E) was blood donation done in the COVID-19 pandemic period. Comparator (C) was a blood donation done in the pre-COVID-19 pandemic period. The outcome (O) assessed was the proportion of the tendency of blood donation.

Study design

The studies included in our meta-analysis were mostly cross-sectional and retrospective. Ethical approval from the institute was not required. 

Search strategy

Following PRISMA guidelines, an extensive search was done by two independent authors (US and SK) across the PubMed, ProQuest, Scopus, and Google Scholar electronic databases. The authors searched only for articles published in English. The free-text words and Medical Subject Heading (MeSH) terms searched in the study were “blood donation” (MeSH term) OR “blood donation” (all fields) AND “COVID-19 pandemic” (MeSH term) OR “impact of COVID-19 pandemic” (all fields) AND “blood donation” (all fields). We also searched from references of screened studies.

Data collection and analysis

Two independent authors (US and SK) independently and rigorously searched eligible articles based on the inclusion and exclusion criteria described above. A third author resolved any discrepancies during data retrieval. Data were extracted independently and entered into a Microsoft Excel sheet by both authors, supervised by another author (AK). We extracted the following data: name of the first author, place of study, period of study, year of publication, number of blood donors in the pre-COVID-19 period and in the COVID-19 pandemic period, and the population of the study region.

Results

**Figure 1:**
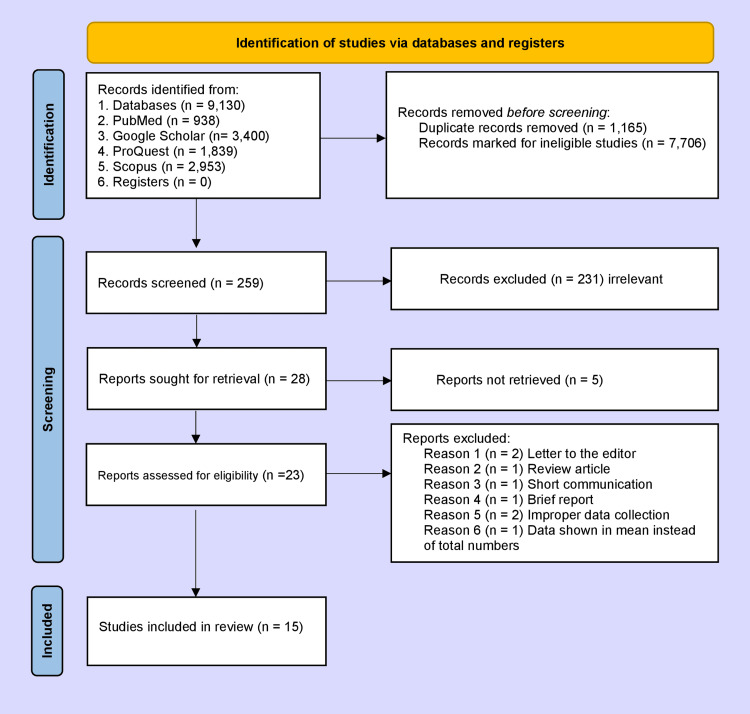
Study flow diagram as per PRISMA 2020. PRISMA: Preferred Reporting Items for Systematic Reviews and Meta-Analysis.

We identified a total of 9,130 studies after searching the different electronic databases, of which 1,165 were duplicate records and 7,706 were ineligible studies, which we removed from our study. On further searching, we excluded an additional 231 irrelevant articles. On further retrieval, we selected 28 studies, of which five were not retrieved. Finally, we selected 15 articles after excluding two letters to the editor [2,3], one review article [1], one short communication [4], and one brief report [5]. In two of the studies [6,7], instead of total blood donation, blood collection per day was shown, and in one study from Saudi Arabia [8], only mean blood collection was shown; therefore, we also excluded these studies (Figure 1). The demographic characteristics of the included studies are given in Table 1.

**Table 1:** Demographic characteristics of the included studies.

Author name	Country of study	Period of study	Study design	Blood donation during pre-COVID-19 period	Blood donation during COVID-19 pandemic period	Population of region during pre-COVID-19 period	Population of region during COVID-19 pandemic period
Raturi and Kusum [9]	India	October 2019 to March 2020	Retrospective descriptive study	1,343	600	11,525,000	11,700,000
Kandasamy et al. [10]	India	April 2020 to June 2020	Cross-sectional observational study	3,888	2,536	68,660,000	69,580,000
Dutt et al. [11]	India	April 2019 to March 2020	Retrospective observational study	20,659	14,596	697,000	702,000
Divya et al. [12]	India	September 2019 to August 2020	Cross-sectional study	2,061	1,174	69,370,000	70,460,000
Ogar et al. [13]	Nigeria	August 2019 to May 2020	Descriptive cross-sectional study	1,244	394	555,000	579,000
Damulak et al. [14]	Nigeria	February 2020 to April 2020	Retrospective descriptive study	3,996	2,954	20,330,000	20,830,000
Grandone et al. [15]	Italy	February 2020 to March 2020	Cross-sectional study	18,100	16,200	4,000,000	4,010,000
Quaglietta et al. [16]	Italy	March 2020 and April 2020	Cross-sectional study	3,143	2,136	319,000	377,000
Wang et al. [17]	China	March 2020 and April 2020	Cross-sectional study	15,609	5,253	57,400,000	64,560,000
Gracia-Erce et al. [18]	Spain	January 2020 to May 2020	Prospective study	34,427	31,563	47,100,000	47,400,000
Politis et al. [19]	Greece	February 2020 to May 2020	Cross-sectional study	7,275	7,407	10,570,000	10,510,000
Tagny et al. [20]	Cameroon	January 2020 to April 2020	Cross-sectional study	9,318	7,292	25,780,000	26,490,000
Dinardo et al. [21]	Brazil	May 2020	Cross-sectional study	19,100	14,322	2,184,000	2,204,000
Yahia [22]	Arabia	September 2019 to May 2020	Cross-sectional study	1,360	1,009	83,000	84,500
Loua et al. [23]	Algeria	January 2020 to May 2020	Cross-sectional study	6,025	4,470	431,000,000	439,000,000
	Angola	January 2020 to May 2020	Cross-sectional study	68,965	49,353	318,000,000	329,000,000
	Benin	January 2020 to May 2020	Cross-sectional study	29,636	25,352	118,000,000	121,000,000
	Burkina Faso	January 2020 to May 2020	Cross-sectional study	45,229	29,391	203,000,000	209,000,000
	Burundi	January 2020 to May 2020	Cross-sectional study	34,237	39,041	115,000,000	119,000,000
	Cameroon	January 2020 to May 2020	Cross-sectional study	47,275	32,328	259,000,000	265,000,000
	Chad	January 2020 to May 2020	Cross-sectional study	9,860	9,903	159,000,000	164,000,000
	Comoros	January 2020 to May 2020	Cross-sectional study	1,106	848	851,000	870,000
	Congo	January 2020 to May 2020	Cross-sectional study	25,998	26,098	538,000,000	551,000,000
	Cole d’lvoire	January 2020 to May 2020	Cross-sectional study	60,201	51,374	257,000,000	264,000,000
	Democratic Republic of Congo	January 2020 to May 2020	Cross-sectional study	255,460	146,693	868,000,000	896,000,000
	Eritrea	January 2020 to May 2020	Cross-sectional study	4,536	4,593	3,490,000	3,550,000
	Eswatini	January 2020 to May 2020	Cross-sectional study	7,185	5,174	1,148,000	1,160,000
	Ethiopia	January 2020 to May 2020	Cross-sectional study	98,340	106,582	1,120,000,000	1,150,000,000
	Gabon	January 2020 to May 2020	Cross-sectional study	9,174	6,999	2,170,000	2,230,000
	Ghana	January 2020 to May 2020	Cross-sectional study	73,063	57,269	304,000,000	311,000,000
	Guinea	January 2020 to May 2020	Cross-sectional study	8,543	6,806	127,000,000	131,000,000
	Kenya	January 2020 to May 2020	Cross-sectional study	59,858	33,419	525,000,000	538,000,000
	Lesotho	January 2020 to May 2020	Cross-sectional study	2,765	2,550	2,125,000	2,140,000
	Madagascar	January 2020 to May 2020	Cross-sectional study	21,868	16,733	269,000,000	277,000,000
	Malawi	January 2020 to May 2020	Cross-sectional study	22,560	21,210	186,000,000	191,000,000
	Mali	January 2020 to May 2020	Cross-sectional study	22,747	16,765	197,000,000	203,000,000
	Mauritania	January 2020 to May 2020	Cross-sectional study	8,017	7,968	4,520,000	4,620,000
	Mauritius	January 2020 to May 2020	Cross-sectional study	19,685	13,727	798,000	1,217,000
	Mozambique	January 2020 to May 2020	Cross-sectional study	54,811	49,207	303,000,000	313,000,000
	Namibia	January 2020 to May 2020	Cross-sectional study	15,204	14,900	2,490,000	2,540,000
	Niger	January 2020 to May 2020	Cross-sectional study	9,619	8,560	233,000,000	242,000,000
	Nigeria	January 2020 to May 2020	Cross-sectional study	9,450	5,879	2,009,000,000	2,061,000,000
	Sao Tome and Principe	January 2020 to May 2020	Cross-sectional study	499	364	215,000	219,000
	Senegal	January 2020 to May 2020	Cross-sectional study	16,442	13,345	162,000,000	167,000,000
	Seychelles	January 2020 to May 2020	Cross-sectional study	675	594	976,000	984,000
	South Africa	January 2020 to May 2020	Cross-sectional study	385,983	384,263	580,000,000	593,000,000
	Togo	January 2020 to May 2020	Cross-sectional study	16,054	16,043	8,080,000	8,280,000
	Uganda	January 2020 to May 2020	Cross-sectional study	122,598	116,856	443,000,000	457,000,000
	United Republic of Tanzania	January 2020 to May 2020	Cross-sectional study	130,038	100,764	580,000,000	597,000,000
	Zambia	January 2020 to May 2020	Cross-sectional study	55,085	46,453	178,000,000	184,000,000
	Zimbabwe	January 2020 to May 2020	Cross-sectional study	41,444	26,899	146,000,000	149,000,000

Study Characteristics

We performed a detailed assessment of the methodological qualities of the selected studies by following the Joanna Briggs Institute (JBI) critical appraisal checklist for systematic reviews and meta-analyses, as shown in Table 2. 

**Table 2:** Methodological quality assessment of potential studies following the JBI critical appraisal checklist. JBI: Joanna Briggs Institute; Y: yes, the article met the criteria; N: no, the articles did not meet some or all of the criteria. *Articles that are excluded due to incompatible metadata.

Author name	Were the criteria for inclusion clearly defined?	Were the study subjects described in detail?	Was the exposure measured in a valid and reliable way?	Were the objective, standard criteria used for the measurement of the condition?	Were the confounding factors identified?	Were strategies to deal with confounding factors stated?	Were the outcomes measured in a valid and reliable way?	Was appropriate statistical analysis done?
Gupta et al. [2]*	Y	N	N	N	Y	Y	N	N
Kasanga et al. [3]*	Y	N	N	N	Y	Y	N	N
Silva-Malta et al. [4]*	Y	N	N	Y	Y	Y	N	N
Vassallo et al. [5]	N	Y	Y	Y	Y	Y	Y	Y
Delbranche et al. [6]*	Y	N	N	Y	Y	Y	Y	Y
Bharat et al. [7]*	Y	N	Y	Y	Y	Y	Y	Y
Hakami et al. [8]*	Y	N	Y	Y	Y	Y	Y	Y
Raturi and Kusum [9]	Y	y	y	y	Y	Y	Y	Y
Kandasamy et al. [10]	Y	Y	Y	Y	Y	Y	Y	Y
Dutt et al. [11]	y	y	y	y	y	y	y	y
Divya et al. [12]	Y	Y	Y	Y	Y	Y	Y	Y
Ogar et al. [13]	Y	Y	Y	Y	Y	Y	Y	Y
Damulak et al. [14]	Y	Y	Y	Y	Y	Y	Y	Y
Grandone et al. [15]	Y	Y	Y	Y	Y	Y	Y	Y
Quaglietta et al. [16]	Y	Y	Y	Y	Y	Y	Y	Y
Wang et al. [17]	Y	Y	Y	Y	Y	Y	Y	Y
Gracia-Erce et al. [18]	Y	Y	Y	Y	Y	Y	Y	Y
Politis et al. [19]	Y	Y	Y	Y	Y	Y	Y	Y
Tagny et al. [20]	Y	Y	Y	Y	Y	Y	Y	Y
Dinadro et al. [21]	Y	Y	Y	Y	Y	Y	Y	Y
Yahia [22]	Y	Y	y	y	y	y	y	y
Loua et al. [23]	y	Y	y	y	y	y	y	y

Out of the 23 articles we selected, all were highly relevant to the study, but one was a systematic review and meta-analysis [1], two were letters to the editor (one by Gupta et al. [2] from India and another by Kasanga et al. [3] from Zambia), and one was a short communication [4]. Two studies, one from France [6] and another from Lucknow, India [7], showed similar data on blood donation, but instead of showing the total number of donations, mean blood donations per day [6] and total blood donations per day [7] were used, respectively, to compare the rate of blood donation in the pre-pandemic and pandemic periods. One study done by Vassallo et al. in the USA was a brief report [5]. The data shown in this study were very relevant but excluded from our study. Another study from Saudi Arabia showed mean donation only instead of total blood donation [8] and was therefore excluded from our study. The studies included in our meta-analysis were done in different countries: four were from India [9-12], two were from Nigeria [13,14], two were from Italy [15,16], and there was one each from China [17], Spain [18], Greece [19], Cameroon [20], Brazil [21], and Saudi Arabia [22]. One study by Loua et al. [23] included data from 37 African countries that are members of the WHO.

Most of the studies were retrospective or cross-sectional. In some studies, blood collection data for an entire country are shown, as in the study of Loua et al. [23], in which data collection was done in 37 African countries, whereas most of the other studies showed data from one or two blood centers.

Although the data collected in the pandemic period were compared with those of the pre-pandemic period, the defined pre-pandemic period was not the same in all studies, as the disease spread across different countries in different months. In one study from China, where the disease occurred first, blood donation during the Spring Festival (i.e., January and February) of 2020 was compared with the Spring Festival of 2019 [17]. In a study by Ogar et al., the pre-pandemic period was October 2019 to February 2020, and the pandemic period was March to May 2020 [13]. Studies by Gracia-Erce et al. and Loua et al. were done from January to May 2020 and compared with those from January to May 2019 [18,23]. A study by Grandone et al. from Italy was done from February to March 2020 and compared with February and March months in 2019 [15]. In another Italian study by Quaglietta et al., data from March and April 2020 were compared with those of March and April 2019 [16]. In four studies from India, the data were collected from different periods. In a study by Kandasamy et al., data from April to June 2020 were compared to those of 2019 [10]. In a study done by Divya et al., data from March to August 2020 were compared with those collected from September 2019 to February 2020 [12]. In another study done by Dutt et al., blood donation data for one-year periods were compared, with April 2020 to March 2021 being compared to April 2019 to March 2020 [11]. In a study by Damulak et al., three-month data periods were compared [14], whereas four-month data periods were compared in a study by Yahia [22], and two-month data periods were compared in a study by Dinardo et al. [21].

A study by Yang et al. from the Zhejiang province of China showed a marked decline in blood donation during the pandemic period; most of the donors in the pre-pandemic period were young and new donors, whereas those in the COVID-19 pandemic period were mostly repeat donors and older [17]. Another study by Ogar et al. [13] from Nigeria showed that most donors were of a young age. One study from Dehradun, India, showed a progressive decrease in blood donation and a similar decrease in blood demand [9]. In one study from Spain, the study period was divided into phases, in which phase I showed a decrease in blood donation, whereas two centers showed an increase in blood donation in phase II [18]. One study from Italy [15] was done over five weeks and compared with the same five weeks of the pre-pandemic period, which found that, initially, there was a progressive decline in blood donation, but later a sharp decline was seen due to lockdown. In most of the studies, voluntary blood donation at camps decreased, and most of the donation was in-house. Furthermore, the number of first-time donors decreased during the pandemic period compared to pre-COVID-19 levels.

**Figure 2:**
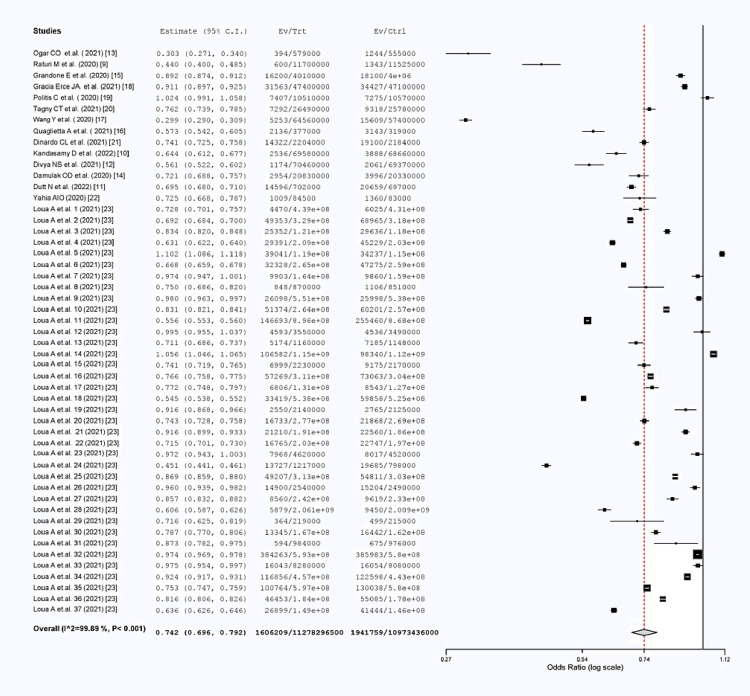
Forest plot showing the pooled difference in the proportion of blood donation between the pre- and post-pandemic periods. The study done by Loua et al. consists of studies on blood donation in 37 WHO countries.

We analyzed the trend in blood donation from before the COVID-19 pandemic to the pandemic period using a forest plot (Figure 2), which showed a 26% decline in blood donation. The largest decline was observed in the study done by Wang [17] from China, which showed a donation decrease of approximately 71% during the pandemic period as compared to before the COVID-19 pandemic. Similar decreases were seen in studies by Raturi and Kusum (66%) [9], Ogar et al. (46%) [13], Divya et al. (44%) [12], Quaglietta et al. (43%) [16], Kandasamy et al. (36%) [10], Dutt et al. (31%) [11], Damulak et al. and Yahia (28%) [14,22], Dinardo et al. (26%) [21], Tagny et al. (24%) [20], Grandone et al. (11%) [15], and Gracia-Erce et al. (9%) [18] during the COVID-19 pandemic period.

A study done by Loua et al. in 37 WHO African countries showed an overall decline of 19% in blood donation [23]. Among the WHO African countries, the greatest decrease in blood donation was seen in Mauritius (50%), followed by Kenya (46%), Democratic Republic of Congo (45%), Cameroon (44%), Nigeria (40%), Burkina Faso (37%), Guinea (33%), Angola (31%), Mali, Eswatini, Sao Tome and Principe (all 29%), Algeria (28%), Madagascar, Gabon (both 26%), United Republic of Tanzania, Comoros (both 25%), Ghana (24%), Senegal (22%), Zambia (19%), Benin, Zimbabwe, Cote d`Ivoire (all 17%), Niger (15%), Mozambique (14%), Seychelles (13%), Lesotho, Malawi (both 9%), and Uganda (8%). Among the WHO African countries, a decrease in blood donation of <5% was seen in Namibia, Mauritania, South Africa, Togo, Congo, and Eritrea [23], which was due to measures taken to combat the pandemic situation. Some countries showed an increase in the number of blood donation, such as Greece (2%), Burundi (10%), and Ethiopia (5%) [19,23], whereas others showed minimal changes in their blood donation patterns.

We used a two-arm proportion analysis as the statistical method to generate the forest plot generation shown in Figure 2. Blood donation during the pre-pandemic period was used as the control for this analysis. We found that the odds ratio was 0.742 (95%CI = 0.696-0.792), and the heterogeneity was 99.89.

Publication bias

**Figure 3:**
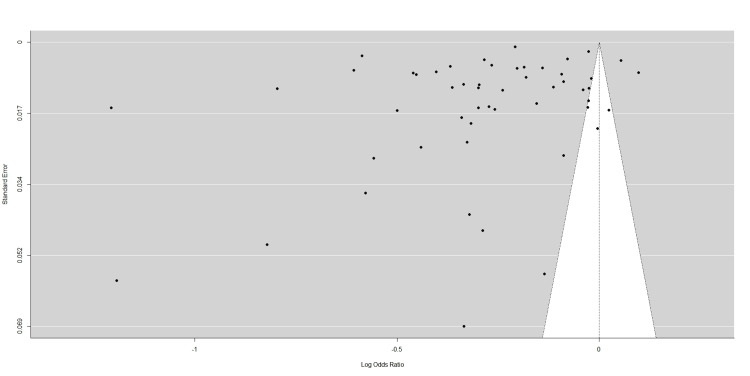
Funnel plot for assessing publication bias.

We investigated the probability of publication bias arising from the published articles using a funnel plot. We observed that there was a significant publication bias (P < 0.001), indicating a high probability of publication bias (Figure 3).

Discussion

The present meta-analysis accurately assessed the consequences of the COVID-19 pandemic on blood donation by using a robust methodology and recommended guidelines. We included a total of 15 studies, which included data from different regions of the world and people of different ethnicities. Severe variance was seen between the studies, which might be due to variability in their sample sizes. In some studies, data were taken from the whole country, whereas others took cumulative data from 37 countries [23]. In some studies, data were collected from one blood center or one regional center. All these factors lead to disparities in sample size.

The reason for the variance was that the data were not collected over similar time periods. Some studies collected data over two-month time intervals, whereas others collected data over a whole year. Another reason for variance was controls in which blood donation was done in the pre-COVID-19 pandemic period and data were collected over different time frames, as the COVID-19 pandemic and lockdown occurred at different times in different countries. The variance was also due to measures taken by some countries to raise blood donation, as the COVID-19 pandemic occurred later, allowing time for these countries to take action to increase blood donation. In some African countries, due to the late country-wide lockdown, the good collaboration between national blood donor associations, civil society organizations, armed and security forces, and national COVID-19 task forces led to either increased blood donation or no or minimal change from the pre-pandemic period [23]. In Greece, as a way to encourage blood donation, regional authorities, municipalities, and voluntary blood donor associations joined hands in a campaign called “All Together We Can,” which resulted in increased blood donation drives with active participation from the general population [19].

Strengths of our study

Previously, a systematic review was published on the impact of COVID-19 on blood donation [1], which included only five studies, of which three [9,15,17] were also included in our study. In the previous meta-analysis [1], none of the studies showed an increase in blood donation during the COVID-19 pandemic, but in our study, two articles covering three countries showed an increase in blood donation for the underlying reasons discussed earlier [19,23]. The previous systematic review included studies published in 2020 only, whereas our study included many recent articles published in 2021 and 2022.

Limitations of our study

We note some severe limitations in our present meta-analysis. Most of the studies were cross-sectional in design, with different periods of data collection. Many studies were not included because they were either not original research articles or they were not published in English. Therefore, data were missing from many countries.

## Conclusions

This study demonstrates that the COVID-19 pandemic has a strong negative impact on blood donation. However, a positive association was seen in countries that started early measures to curtail the infection and increase blood donation by forming policies in collaboration with social organizations, governments, and security forces. The WHO and all countries should form a policy in advance to combat any such condition in the future so that no one suffers from a shortage of blood.
